# Voltage-Dependent Rhythmogenic Property of Respiratory Pre-Bötzinger Complex Glutamatergic, Dbx1-Derived, and Somatostatin-Expressing Neuron Populations Revealed by Graded Optogenetic Inhibition^1^,^2^,^3^

**DOI:** 10.1523/ENEURO.0081-16.2016

**Published:** 2016-06-03

**Authors:** Hidehiko Koizumi, Bryan Mosher, Mohammad F. Tariq, Ruli Zhang, Naohiro Koshiya, Jeffrey C. Smith

**Affiliations:** Cellular and Systems Neurobiology Section, National Institute of Neurological Disorders and Stroke, National Institutes of Health, Bethesda, Maryland 20892

**Keywords:** brainstem microcircuit, breathing, optogenetics, rhythmogenesis

## Abstract

The rhythm of breathing in mammals, originating within the brainstem pre-Bötzinger complex (pre-BötC), is presumed to be generated by glutamatergic neurons, but this has not been directly demonstrated. Additionally, developmental expression of the transcription factor Dbx1 or expression of the neuropeptide somatostatin (Sst), has been proposed as a marker for the rhythmogenic pre-BötC glutamatergic neurons, but it is unknown whether these other two phenotypically defined neuronal populations are functionally equivalent to glutamatergic neurons with regard to rhythm generation. To address these problems, we comparatively investigated, by optogenetic approaches, the roles of pre-BötC glutamatergic, Dbx1-derived, and Sst-expressing neurons in respiratory rhythm generation in neonatal transgenic mouse medullary slices *in vitro* and also more intact adult perfused brainstem-spinal cord preparations *in situ*. We established three different triple-transgenic mouse lines with Cre-driven Archaerhodopsin-3 (Arch) expression selectively in glutamatergic, Dbx1-derived, or Sst-expressing neurons for targeted photoinhibition. In each line, we identified subpopulations of rhythmically active, Arch-expressing pre-BötC inspiratory neurons by whole-cell recordings in medullary slice preparations *in vitro*, and established that Arch-mediated hyperpolarization of these inspiratory neurons was laser power dependent with equal efficacy. By site- and population-specific graded photoinhibition, we then demonstrated that inspiratory frequency was reduced by each population with the same neuronal voltage-dependent frequency control mechanism in each state of the respiratory network examined. We infer that enough of the rhythmogenic pre-BötC glutamatergic neurons also have the Dbx1 and Sst expression phenotypes, and thus all three phenotypes share the same voltage-dependent frequency control property.

## Significance Statement

The brainstem pre-Bötzinger complex (pre-BötC) is the origin of rhythmic neural activity producing inspiratory movements in mammals. Despite over two decades of investigation, there is the central problem of establishing the causal role of specific pre-BötC neuron populations in generating this rhythmic motor behavior. By optogenetic and electrophysiological approaches, we reveal that excitatory glutamatergic, transcription factor Dbx1-derived, and somatostatin-expressing neuronal populations, overlapping within the pre-BötC region, are functionally equivalent in respiratory rhythm generation with the same neuronal voltage-dependent frequency control mechanism in different functional and developmental states of the respiratory network, including neonatal *in vitro* and adult *in situ* conditions. Our results help resolve the long-standing problem of delineating the relative roles of these excitatory interneuron populations in rhythm generation.

## Introduction

Brainstem and spinal circuits generating rhythmic motor behaviors in mammals, such as breathing and locomotion, consist of interacting excitatory and inhibitory interneurons, with glutamatergic neurons proposed to form a core component of the rhythm-generating mechanisms within these motor circuits. Establishing the causal roles and intrinsic mechanisms of these excitatory neurons is one of the central problems in defining the origins of rhythmic motor behavior. In the present study, we have analyzed these roles/mechanisms in the pre-Bötzinger complex (pre-BötC), a localized region of the ventrolateral medulla (Smith et al., 1991; Feldman et al., 2013) that is known to be critical for generating the normal rhythmic respiratory neural pattern *in vivo* (Ramirez et al., 1998; Gray et al., 2001; Wenninger et al., 2004; Richter and Smith, 2014). The pre-BötC represents a functionally defined microcircuit with excitatory mechanisms that can provide an understanding of rhythmogenesis in mammalian motor and potentially other neural systems.

Optical imaging-based (Koshiya and Smith, 1999; Gourévitch and Mellen, 2014), electrophysiological (Del Negro et al., 2005; Koizumi and Smith, 2008), developmental (Bouvier et al., 2010; Gray et al., 2010), and computational modeling analyses (Rubin et al., 2009; Rybak et al., 2014) have proposed that pre-BötC glutamatergic neurons constitute the rhythmogenic kernel generating oscillatory drive to produce inspiratory movements. However, glutamatergic neurons have not been shown to be the critical neuronal kernel for respiratory rhythm generation, because the genetic-based tools to selectively target, manipulate activity, and examine the functional properties of this population have not yet been applied. We show the critical requirement for glutamatergic neurons by optogenetic approaches in transgenic (Tg) mice with Cre-driven expression of Archaerhodopsin-3 (Arch; light-driven proton pump; Chow et al., 2010) in these neurons. This approach enabled temporally controlled, graded attenuation of activity and loss of function of pre-BötC glutamatergic neurons, with site specificity, in a manner consistent with a neuronal voltage-dependent rhythmogenic mechanism. This result was obtained in the following three different states of the pre-BötC network: (1) isolated in rhythmically active neonatal medullary slices *in vitro*; (2) embedded in more intact brainstem respiratory circuits in arterially perfused adult “*in situ*” brainstem-spinal cord preparations; and (3) in slice-like reduced states of the adult *in situ* system (Smith et al., 2007) where intrinsic rhythmogenic mechanisms could also be effectively probed.

Surrogate phenotypes for pre-BötC glutamatergic neurons, specifically the expression of somatostatin (Sst) peptide (Stornetta et al., 2003b; Tupal et al., 2014; Sherman et al., 2015), or descendance from progenitor cells expressing the developmentally transient transcription factor Dbx1 (Bouvier et al., 2010; Gray et al., 2010), have been used for presumptive targeting of glutamatergic neurons for cell type-specific loss-of-function experiments. Pharmacogenetic inhibition of Sst-expressing pre-BötC neurons in adult rats *in vivo* (Tan et al., 2008) or photoablation of Dbx1-derived pre-BötC neurons in neonatal mouse slices *in vitro* (Wang et al., 2014) disrupts inspiratory rhythm generation. However, it is currently unknown whether these neuronal sets are mechanistically equivalent to glutamatergic neurons in rhythm generation. In addition, it has not yet been demonstrated that Sst-expressing neurons are rhythmically active and have cellular properties consistent with a rhythmogenic function. Furthermore, the role of Sst-expressing neurons in rhythm generation has been questioned (Tupal et al., 2014). Dbx1-derived pre-BötC neurons, many of which were found to be glutamatergic from histochemical analyses (Gray, 2013), and rhythmically active in neonatal mouse medullary slices *in vitro* (Picardo et al., 2013), are also not exclusively respiratory pre-BötC glutamatergic neurons. Like Sst-expressing neurons, Dbx1-derived neurons represent a heterogeneous superset of neurons, including inhibitory neurons within and outside of the pre-BötC, distributed widely in the reticular formation. Given the importance ascribed to these two neuronal phenotypes, we also addressed the important question in the field of whether they have rhythmogenic properties similar to those found here for pre-BötC glutamatergic neurons. This was accomplished by comparatively analyzing light-induced perturbations and photoinhibition of respiratory rhythm in VgluT2-Cre-Arch mice versus Cre-driver mouse strains specific for Dbx1-derived or Sst-expressing neurons with Cre-dependent Arch expression. In addition to this functional analysis, we probed the overlapping of these phenotypes in our transgenic lines by reporter fluorescent protein expression and immunohistochemical labeling. We demonstrate that glutamatergic, Dbx1-derived, and Sst-expressing neuronal populations, which we confirmed anatomically overlap within the pre-BötC, are equally effective, with a quantitatively identical neuronal voltage-dependent mechanism, in attenuating and disrupting rhythm generation by site-specific, graded photoinhibition. These results were obtained in the different functional and developmental states of the respiratory network examined, including the neonatal transgenic mouse *in vitro* and adult *in situ* conditions, supporting our general conclusion that these neurons are functionally/mechanistically equivalent in rhythm generation.

## Materials and Methods

### Animal procedures

All animal procedures were approved by the Animal Care and Use Committee of the National Institute of Neurological Disorders and Stroke.

### Cre-dependent double/triple-transgenic mouse experimental models

For comparative studies of population-specific roles of pre-BötC glutamatergic, Dbx1-derived, and Sst-expressing neurons in respiratory rhythm generation, we established Cre-dependent double- or triple-Tg mouse models. To study glutamatergic neuron function, we used Slc17a6^tm2(cre)Lowl^ knock-in Cre-driver mice lines (VgluT2-ires-Cre; obtained from The Jackson Laboratory), in which Cre-recombinase activity has been verified in glutamatergic VgluT2-positive neuron cell bodies (Vong et al., 2011). We also used Dbx1^tm1.1(cre)Mull^ Cre-driver mouse lines (Dbx1-ires-Cre; obtained from the Mutant Mouse Resource and Research Center) to study Dbx1-derived neurons, and Sst^tm2.1(cre)Zjh^ knock-in Cre-driver mice lines (Sst-ires-Cre) for Sst-expressing neurons (The Jackson Laboratory). Each Cre-driver mouse line was crossed with a Cre-dependent reporter strain (B6.Cg-Gt(ROSA)26Sor^tm9(CAG-tdTomato)Hze^, Rosa-CAG-LSL-tdTomato-WPRE, The Jackson Laboratory) to obtain offspring with the expression of red fluorescent protein variant tdTomato in Cre-expressing neurons. These double-Tg lines (VgluT2-tdTomato, Dbx1-tdTomato, and Sst-tdTomato) were analyzed histologically and subsequently used to produce triple-Tg lines (below) for optogenetic experiments. The Cre-driver lines were also crossed with a Cre-dependent optogenetic mouse strain (B6;129S-Gt(ROSA)26Sor^tm35.1(CAG-aop3/GFP)Hze^, Rosa-CAG-LSL-Arch-GFP-WPRE, The Jackson Laboratory) to generate double-Tg offspring (VgluT2-Arch-GFP, Dbx1-Arch-GFP, and Sst-Arch-GFP) with expression of Arch–green fluorescent protein (GFP) fusion protein in Cre-expressing neurons that were initially used to verify the efficacy of Cre-driven Arch-GFP expression. For optogenetic experiments, Rosa-CAG-LSL-Arch-GFP-WPRE mice were crossed with VgluT2-tdTomato, Dbx1-tdTomato, or Sst-tdTomato mice to establish three triple-Tg mouse models: VgluT2-tdTomato-Arch-GFP, Dbx1-tdTomato-Arch-GFP, and Sst-tdTomato-Arch-GFP mouse lines, enabling specific photoinhibition of each of the three genetically targeted neuron populations in separate experiments as well as imaging these populations in slice electrophysiology experiments *in vitro*.

For neurotransmitter phenotype analyses of pre-BötC neurons (below), we established another triple-Tg mouse line (VgluT2-tdTomato-GAD67-GFP) by crossing VgluT2-tdTomato with a GAD67-GFP knock-in mouse line (Tamamaki et al., 2003) to obtain offspring with the expression of tdTomato in VgluT2-positive, glutamatergic neurons, and GFP in GAD67-positive, GABAergic neurons.

### Immunohistochemical labeling of pre-BötC neurons

Fluorescence immunolabeling with Sst antibody was used to identify Sst expression in VgluT2-, Dbx1-, and Sst-tdTomato neurons bilaterally within the pre-BötC in, respectively, immersion or transcardially perfusion fixed tissue sections from neonatal and mature VgluT2-tdTomato, Dbx1-tdTomato, or Sst-tdTomato double-Tg strains. Fluorescence immunolabeling with glutaminase (GLS2) antibody was used to assess consistency with a glutamatergic phenotype of VgluT2-, Dbx1-, and Sst-positive pre-BötC neurons. In addition, we immunoreacted tissue from the VgluT2-tdTomato-GAD67-GFP triple-Tg mouse line with glycine antibody and also Sst antibody in experiments to differentiate GABAergic and glycinergic neurons from VgluT2- and Sst-expressing pre-BötC neurons. The medulla oblongatas obtained from all of these mice were fixed in 4% paraformaldehyde (w/v) in PBS, cryoprotected overnight at 4°C in a 30% sucrose, 0.1 m PBS solution, and sectioned coronally or parasagittally at 30 or 50 µm with a freezing microtome. For fluorescence immunohistochemistry, floating sections were incubated with 10% donkey serum in PBS with Triton X-100 (0.3%) and subsequently were incubated for 48–72 h at room temperature with primary antibodies for Sst (rabbit anti-Sst, 1:500; catalog #T-4103, Peninsula Laboratories; Tan et al., 2008), glutaminase (rabbit anti-glutaminase, 1:1000; catalog #ab113509, Abcam), glycine (rabbit anti-glycine, 1:5000; catalog #IG1001, ImmunoSolution), and choline acetyltransferase (ChAT; goat anti-ChAT, 1:200; catalog #AB144P, EMD Millipore) to label motoneurons. Individual sections were then rinsed with PBS and incubated for 2 h with secondary antibodies (donkey anti-rabbit Alexa Fluor 647, 1:500 for Sst labeling; donkey anti-rabbit Dylight 488, 1:500 for glutaminase; donkey anti-rabbit Dylight 405, 1:500 for glycine; donkey anti-goat Alexa Fluor 488, 1:500 for ChAT; Jackson ImmunoResearch). Individual sections were mounted on slides and covered with an anti-fading medium (Fluoro-Gel, Electron Microscopy Sciences). Fluorescent labeling of neurons was visualized with a laser-scanning confocal imaging system (model LSM 510, Zeiss). As a negative control for immunoreactivity, we obtained fluorescent images from slices treated with secondary but not primary antibodies. For tallying the number of Sst- and glutaminase-labeled pre-BötC neurons, as presented in Results, we counted labeled neurons within a region (300–400 µm in diameter, depending on animal size) encompassing the pre-BötC on each side of the medulla in every other 30-µm-thick section through this region. All images were color/contrast enhanced and adjusted with a thresholding filter in Adobe Photoshop.

### Rhythmically active medullary slice preparations *in vitro*

We performed combined optogenetic and electrophysiological experiments with rhythmically active *in vitro* slice preparations (250- to 350-µm-thick transverse slices) cut from the medulla oblongatas from neonatal [postnatal day 4 (P4) to P8] transgenic mice of either sex. These slices contain the active bilateral pre-BötC, premotor circuits transmitting rhythmic inspiratory activity, and rostral end of the hypoglossal motor nucleus (XII) with intact XII nerve rootlets for recording inspiratory motor output (Koizumi et al., 2013). The slice was superfused (4 ml/min) in a recording chamber (0.4 ml) with artificial CSF (ACSF) containing the following (in mm): 124 NaCl, 25 NaHCO_3_, 3 KCl, 1.5 CaCl_2_, 1.0 MgSO_4_, 0.5 NaH_2_PO_4_, and 30 d-glucose equilibrated with 95% O_2_ and 5% CO_2_, pH 7.35–7.40 at 27°C. During experiments, rhythmic respiratory activity in the pre-BötC and XII nerves was maintained by elevating the superfusate K^+^ concentration to 8–9 mm.

### Arterially perfused mouse brainstem-spinal cord preparations *in situ*

For experiments performed with *in situ* arterially perfused brainstem-spinal cord preparations from mature Tg mice of either sex (weight, 20-30 g; age, 3–5 months) with rhythmically active brainstem and spinal respiratory circuits (Paton, 1996; Smith et al., 2007), preheparinized (1000 units, i.p.) mice were anesthetized deeply with 5% isoflurane until loss of the paw withdrawal reflex, and the portion of the body caudal to the diaphragm was removed. The head and thorax were immersed in ice-chilled carbogenated ACSF solution containing the following (in mm): 1.25 MgSO_4,_ 1.25 KH_2_PO_4_, 5.0 KCl, 25 NaHCO_3_, 125 NaCl, 2.5 CaCl_2_, 10 dextrose, and 0.1785 polyethylene glycol. The brain was decerebrated at a precollicular level, and the descending aorta, thoracic phrenic nerve (PN), and cervical vagus nerves were surgically isolated. The dorsal brainstem was exposed by craniotomy and cerebellectomy. The preparation was transferred to a recording chamber and secured in a stereotaxic head frame with the dorsal side up. The descending aorta was cannulated with a double-lumen catheter (DLR-4, Braintree Scientific) for ACSF perfusion with a peristaltic roller pump (catalog #505D, Watson-Marlow) and for the recording of perfusion pressure with a pressure transducer. The ACSF perfusate was gassed with 95% O_2_-5% CO_2_ and maintained at 31°C. Vecuronium bromide or rocuronium bromide (2-4 µg/ml; SUN Pharmaceutical Industries) was added to the perfusate to block neuromuscular transmission. Throughout the experiments, the perfusion pressure was maintained at between 70 and 80 mmHg (Pickering and Paton, 2006) with vasopressin application (200–400 pm as required; APP Pharmaceuticals) and by adjusting the perfusion pump speed to avoid the possible effects of pressure changes on respiratory activity.

We studied the following two types of adult mouse *in situ* preparations (see Fig. 11): (1) the “intact” *in situ* preparation, which preserves circuit interactions including between the pons, retrotrapezoid nucleus (RTN), BötC, pre-BötC, and rostral ventral respiratory group (rVRG) required to generate a normal three-phase rhythmic respiratory pattern/motor output, and (2) the “reduced” *in situ* preparation, in which the excitation state of pre-BötC circuits was reduced by removing pontine and RTN excitatory inputs via transection at the rostral boundary of the pre-BötC that generates in the adult system a reduced, “slice-like” pattern with one (active) phase of rhythmic inspiratory motor output (Smith et al., 2007).

### Electrophysiological recording

To monitor respiratory network activity and motor output, we recorded, with fire-polished glass suction electrodes, inspiratory activity from XII nerves in the *in vitro* slice preparations, and inspiratory and postinspiratory activity from cervical vagus nerves and inspiratory activity from phrenic nerves in the *in situ* perfused brainstem-spinal cord preparations, as described previously (Smith et al., 2007). Electrophysiological signals were amplified (50,000–100,000×; CyberAmp 380, Molecular Devices), band-pass filtered (0.3–2 kHz), digitized (10 kHz) with an analog-to-digital (AD) converter [PowerLab, ADInstruments, or Cambridge Electronics Design (CED)], and then rectified and integrated digitally with Spike 2 software (CED). Extracellular population activity from pre-BötC respiratory neurons in the perfused *in situ* preparations was also recorded with a fine glass pipette electrode (resistance, 5-8 MΩ) filled with 0.5 m NaAc (Sigma-Aldrich). All extracellular recordings were stable throughout the experiments. Voltage- and current-clamp data from slice experiments were recorded with an EPC-10 patch-clamp amplifier (HEKA Electronics) controlled by PatchMaster software (HEKA; 2.9 kHz low-pass filter, sampled at 100 kHz). Whole-cell recording electrodes (borosilicate glass pipette, 4–6 MΩ), positioned with microdrives (Scientifica), contained the following (in mm): 130.0 K-gluconate, 5.0 Na-gluconate, 3.0 NaCl, 10.0 HEPES, 4.0 Mg-ATP, 0.3 Na-GTP, and 4.0 sodium phosphocreatine, pH 7.3 adjusted with KOH. In all cases, measured potentials were corrected for the liquid junction potential (−10 mV). Series resistance was compensated on-line by ≥80%, and the compensation was periodically readjusted. Neurons exhibiting clear evidence of poor space clamp, such as unclamped action potential currents during voltage-clamp measurements, were excluded from the analysis.

### Imaging and functional identification of pre-BötC respiratory neurons *in vitro*

For neuronal electrophysiology *in vitro*, we imaged tdTomato-labeled VgluT2-, Dbx1- or Sst-positive pre-BötC neurons as well as Arch-GFP fusion protein in the cell membranes in the VgluT2-tdTomato-Arch-GFP, Dbx1-tdTomato-Arch-GFP, and Sst-tdTomato-Arch-GFP triple-Tg mouse slices (for examples, see Fig. 2). We performed whole-cell patch-clamp recording from these neurons to functionally identify pre-BötC VgluT2-, Dbx1- or Sst-expressing inspiratory neurons (see Figs. 6, 7) and to measure light-induced hyperpolarization. In some experiments, we also imaged multineuronal rhythmic activity with Ca^2+^-sensitive dye [Oregon Green BAPTA-1 AM (OGB), Invitrogen] loaded into neurons >1 h after local microinjection of OGB in the pre-BötC (Koizumi et al., 2013). Live activity imaging in all cases was performed with a two-photon laser-scanning upright microscope (TCS SP5 II MP with DM6000 CFS system, LAS AF software, a 20× water objective, numerical aperture 1.0, Leica; and 560 nm beam splitter, emission filter 525/50, Semrock). A Ti:sapphire pulsed laser (MaiTai, Spectra-Physics) was used at 800-880 nm with DeepSee predispersion compensation. The Ca^2+^-dye fluorescence imaging was performed with the tandem resonant scanner (16 kHz bidirectional, ∼25 frames/s for a 512 × 512 pixel scan), and scan range magnifications of 1.7× or 3× were typically used, achieved by scan range modifications covering a 434 or 246 µm square, respectively, containing/within the pre-BötC region. The infrared excitation laser for two-photon fluorescence was simultaneously used for transmission bright-field illumination to achieve a Dodt gradient contrast structural imaging to give fluorescence and structural images matched to pixels, allowing us to accurately place a patch pipette on tdTomato-labeled neurons (see Fig. 6*B*) and to functionally identify pre-BötC inspiratory neurons, which were active in phase with XII inspiratory network activity (see Figs. 6*C*,*D*, 7). For Ca^2+^ dynamic imaging of rhythmic inspiratory activity, fluorescence images were acquired in real time along with electrophysiological signals of inspiratory XII activity (LAS AF, Electrophysiology Module version 2.60; see Fig. 7).

### Optogenetic inhibition of pre-BötC neurons

Laser illumination for optogenetic experiments was performed with an orange laser (continuous 593 nm illumination; OptoDuet Laser, IkeCool). Laser power (2–15 mW) was measured with an optical power and energy meter (PM100D, ThorLabs). Illumination epochs were controlled by a pulse stimulator (Master-8, A.M.P.I.). Optical fibers, from a bifurcated fiberoptic patch cable, each terminated by an optical cannula (100 µm diameter, ThorLabs), were positioned unilaterally or bilaterally on the surface of the pre-BötC in the *in vitro* rhythmically active slice preparations. In the *in situ* brainstem-spinal cord preparations, the same fine fiber optics were bilaterally implanted to a depth just dorsal to the pre-BötC. Positioning of the optical fibers by micromanipulators was based on predetermined coordinates for the pre-BötC from extracellular recordings of respiratory neurons within the ventral respiratory column, and were further confirmed with *post hoc* histology. We analyzed light-induced perturbations of the frequency and amplitude of rhythmic inspiratory activity/motor output as well as durations of inspiratory and expiratory phases. We conducted control experiments in *in vitro* slices and *in situ* preparations from the VgluT2-, Dbx1-, and Sst-tdTomato non-Arch-expressing Tg mouse strains (*n* = 2 each) to test for photoinduced perturbations of frequency, phase durations, or inspiratory burst amplitude, and also used for statistical comparisons with data from the VgluT2-, Dbx1-, and Sst-tdTomato-Arch-GFP Tg mouse strains. Site specificity of the perturbations was examined by systematically repositioning the optical cannula bilaterally in reticular formation sites immediately rostral or caudal to the pre-BötC region.

### Quantification of respiratory parameters and statistics

Respiratory parameters were quantified off-line with custom algorithms using IDL (Exelis Visual Information Solutions) on AD converted data. Inspiratory peak times were defined using derivatives of smoothed rectified XII nerve activity from *in vitro* preparations and PN activity from *in situ* preparations, from which instantaneous inspiratory burst frequency was calculated. For the statistical analyses, the inspiratory burst frequency during laser application was normalized to the preillumination control frequency calculated as an average from 20 inspiratory bursts before laser application. The interburst (expiratory phase) baseline value was defined using a median filter and was applied to quantify the XII or PN amplitude by subtraction for every burst. Times of XII and PN signals crossing the threshold (normally 20% of the amplitude) were used to define onsets and offsets of XII or PN inspiratory activity, inspiratory phase duration (*T*_I_) and expiratory phase duration (*T*_E_). To obtain representative time courses, values of these parameters for each cycle were smoothed by a moving box median filter. Time series data as well as summary graphs were plotted with Igor Pro (WaveMetrics). Statistical significance (*p* < 0.01) was determined (Table 1) with a two-tailed Student’s *t* test when comparing two groups, and one-way ANOVA for comparing multiple groups in conjunction with *post hoc* Tukey’s HSD test for pairwise comparison. Summary data are presented as the mean ± SD.

**Table 1:** Summary of statistics from figures

Figure	Data structure	Type of test	*p* value
6E (xhalf)	Normally distributed	Student’s *t* test	0.904
6E (slope)	Normally distributed	Student’s *t* test	0.938
8 (xhalf)	Normally distributed	One-way ANOVA	0.640
8 (slope)	Normally distributed	One-way ANOVA	0.480
10A	Normally distributed	One-way ANOVA	0.499
10B	Normally distributed	One-way ANOVA	0.182
10C	Normally distributed	One-way ANOVA	0.512
10D	Normally distributed	One-way ANOVA	0.448
13A	Normally distributed	One-way ANOVA	0.246
13B	Normally distributed	One-way ANOVA	0.568
13C	Normally distributed	One-way ANOVA	0.288
13D	Normally distributed	One-way ANOVA	0.462
15A	Normally distributed	One-way ANOVA	0.564
15B	Normally distributed	One-way ANOVA	0.649
15C	Normally distributed	One-way ANOVA	0.594
15D	Normally distributed	One-way ANOVA	0.589
16B			
Frequency, 2 mW	Normally distributed	Student’s *t* test	0.891
Frequency, 5 mW	Normally distributed	Student’s *t* test	0.482
Frequency, 10 mW	Normally distributed	Student’s *t* test	0.607
Amplitude, 2 mW	Normally distributed	Student’s *t* test	0.016
Amplitude, 5 mW	Normally distributed	Student’s *t* test	0.0012
Amplitude, 10 mW	Normally distributed	Student’s *t* test	0.00017

## Results

### Cre-dependent tdTomato reporter neuronal labeling and Arch-GFP expression in the pre-BötC region in triple-transgenic mice

**Figure 1.**
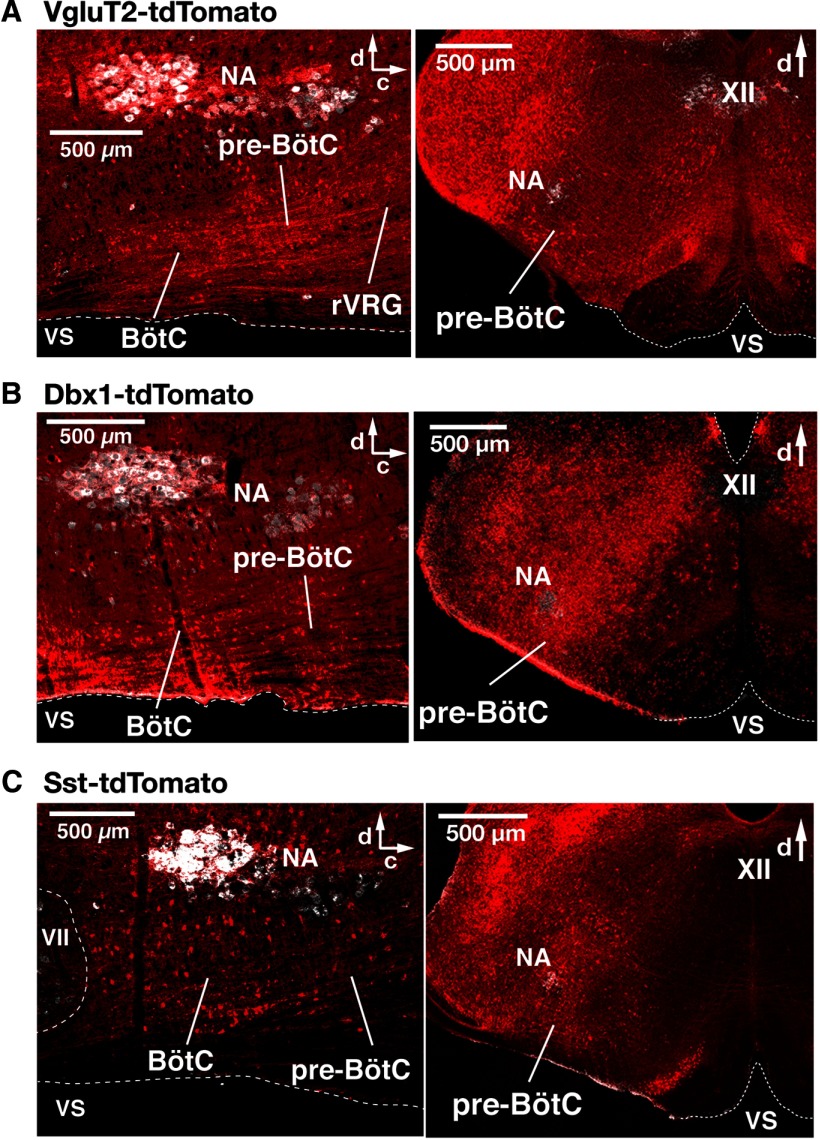
Spatial distribution of Cre-dependent tdTomato-labeled neurons in the medullary reticular formation in VgluT2-tdTomato, Dbx1-tdTomato, and Sst-tdTomato double-Tg mouse strains. ***A–C***, Confocal fluorescence microscopy images of parasagittal sections at the level of nucleus ambiguus (NA) and coronal sections at the level of the pre-BötC from adult VgluT2-tdTomato (***A***), Dbx1-tdTomato (***B***), and Sst-tdTomato (***C***) mice, representing an overview of distributions of Cre-dependent tdTomato-labeled neurons (red). Motoneurons of NA and the XII immunostained with ChAT antibody (white) provide reference landmarks for levels of the medulla represented in the sections. The parasagittal sections illustrate the extensive distribution of tdTomato-labeled neurons throughout the ventral medullary reticular formation, including within the pre-BötC, the BötC, and the rVRG. Coronal sections illustrate that tdTomato-labeled neurons are distributed within the pre-BötC, and adjacent regions of the reticular formation (RF), including the intermediate RF dorsomedial to NA, where hypoglossal premotor neurons are located. VS, Ventral surface; d, dorsal; c, caudal.

**Figure 2.**
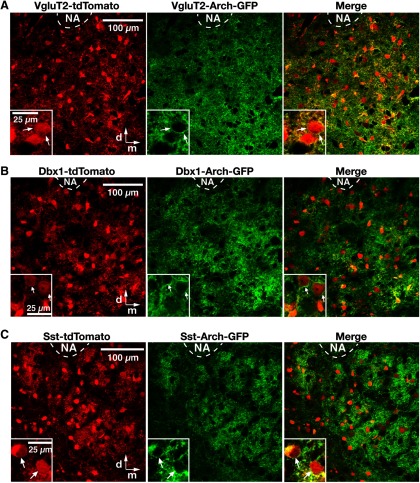
Cre-dependent tdTomato reporter neuronal labeling and Arch-GFP expression in the pre-BötC region in triple-transgenic mice. ***A–C***, Two-photon LSM single optical plane “live” images of the pre-BötC region ventral to nucleus ambiguus (NA) on one side of the medulla in an *in vitro* neonatal medullary slice from the VgluT2-tdTomato-Arch-GFP (***A***), Dbx1-tdTomato-Arch-GFP (***B***), and Sst-tdTomato-Arch-GFP (***C***) triple-Tg mouse lines, presenting an overview of distributions of Cre-conditional tdTomato reporter protein labeling (red) and Arch-GFP fusion protein expression (green) of neurons throughout the pre-BötC region. Insets, Higher-magnification images illustrating neurons with Arch-GFP expression in somal membranes, as confirmed in the merged image for tdTomato-labeled VgluT2-expressing, Dbx1-derived, and Sst-expressing pre-BötC neurons. All images have the same dorsomedial anatomical orientation. d, Dorsal; m, medial.

All optogenetic experiments were performed with Cre-conditional VgluT2-tdTomato-Arch-GFP, Dbx1-tdTomato-Arch-GFP, and Sst-tdTomato-Arch-GFP triple-Tg mouse lines. We histologically validated the Cre-driver Tg lines by verifying, via confocal microscopy in fixed serial sections, Cre-dependent tdTomato neuronal labeling in the pre-BötC initially in double-Tg lines (see Materials and Methods; Fig. 1), and subsequently Cre-driven Arch expression was validated by tdTomato and Arch-GFP coexpression in the triple-Tg lines within the pre-BötC in live *in vitro* neonatal medullary slices imaged by two-photon laser-scanning microscopy (LSM; *n* = 6 for each line; Fig. 2), as well as in fixed serial sections from neonatal and adult mice by confocal microscopy (*n* = 3 for each Tg line). We confirmed extensive tdTomato- and Arch-GFP colabeled neurons in the VgluT2-tdTomato-Arch-GFP (Fig. 2*A*), Dbx1-tdTomato-Arch-GFP (Fig. 2*B*), or Sst-tdTomato-Arch-GFP (Fig. 2*C*) lines throughout the entire pre-BötC region bilaterally. Arch-GFP fusion protein was heavily expressed in processes and somal membranes of tdTomato-labeled neurons throughout the pre-BötC in all cases. We also note that neuronal tdTomato and Arch-GFP expression was distributed extensively throughout the medullary reticular formation, including in ventrolateral medullary respiratory regions adjacent to the pre-BötC (i.e., in the rVRG caudal to, and in the BötC region rostral to the pre-BötC; Fig. 1, examples of the distribution of Cre-driven tdTomato labeling).

### Validation of VgluT2-Cre line and glutamatergic phenotype of Dbx1-derived and Sst-expressing pre-BötC neurons

**Figure 3.**
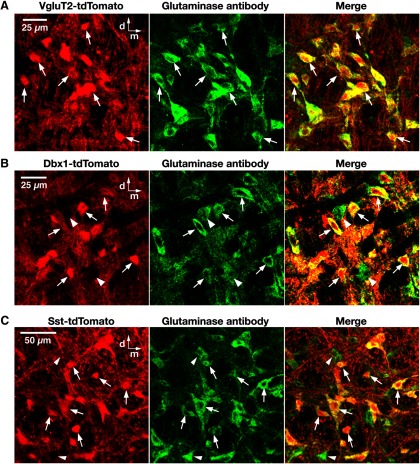
Validation of VgluT2-Cre line and glutamatergic phenotype of Dbx1-derived and Sst-expressing pre-BötC neurons. ***A***, Confocal fluorescence microscopy images of a subregion within the pre-BötC in a fixed coronal section from VgluT2-tdTomato double-Tg strains, showing Cre-dependent tdTomato labeling (red) and immunolabeling with glutaminase antibody (green) in pre-BötC neurons. Merged image illustrates that essentially all of the Cre-mediated tdTomato-labeled VgluT2 neurons coexpress glutaminase, which is consistent with a glutamatergic phenotype of these neurons. ***B***, ***C***, Confocal fluorescence microscopy images of a pre-BötC subregion in a fixed coronal section from Dbx1-tdTomato (***B***) and Sst-tdTomato (***C***) double-Tg strains, showing neuronal tdTomato labeling (red) and immunolabeling with glutaminase antibody (green). Merged image illustrates tdTomato-labeled Dbx1-derived (***B***) and Sst-expressing (***C***) pre-BötC neurons that coexpress glutaminase (arrows). Examples of glutaminase antibody-labeled neurons without somal tdTomato expression are also indicated (arrowheads). All images have the same dorsomedial anatomical orientation. d, Dorsal; m, medial.

**Figure 4.**
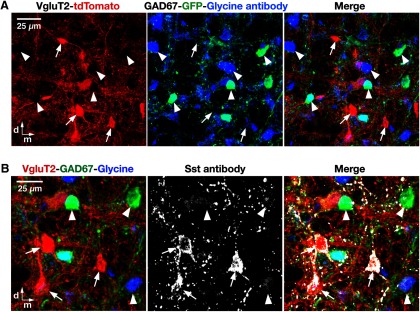
Glutamatergic phenotype of VgluT2- and Sst-expressing pre-BötC neurons in the VgluT2-tdTomato-GAD67-GFP triple-transgenic mouse line. ***A***, Confocal images of a subregion within the pre-BötC in a fixed coronal section from the VgluT2-tdTomato-GAD67-GFP triple-Tg strain, showing Cre-dependent VgluT2-tdTomato neuronal labeling (red), GAD67-knock-in GFP neuronal labeling (green), and immunolabeling with glycine antibody (blue) in the pre-BötC neurons. Merged image confirms no overlap of tdTomato neuronal somal labeling (examples indicated by arrows) with GAD67-GFP or glycine antibody expression (arrowheads), except for labeling of terminals in some cases, also verifying that all the Cre-driven tdTomato-labeled VgluT2 neurons are glutamatergic neurons. ***B***, Confocal images of a pre-BötC subregion in an adult VgluT2-tdTomato-GAD67-GFP mouse, illustrating Cre-dependent VgluT2-tdTomato neuronal labeling (red), GAD67-knock-in GFP neuronal labeling (green), immunolabeling with glycine antibody (blue), and Sst antibody labeling (white) of pre-BötC neurons. Merged image illustrates that tdTomato-labeled VgluT2 neurons coexpress Sst (arrows), but GAD67-GFP- or glycine antibody-positive neurons do not express Sst (arrowheads). All images have the same dorsomedial anatomical orientation. d, Dorsal; m, medial.

We further validated the VgluT2-Cre line by glutaminase antibody labeling. Nearly all the tdTomato-labeled pre-BötC neurons examined (98.5%, *n* = 703 of 714 neurons counted bilaterally from three mice) coexpressed glutaminase (Fig. 3*A*), which is consistent with a glutamatergic phenotype. We also confirmed this phenotype of VgluT2-expressing neurons by using a VgluT2-tdTomato-GAD67-GFP triple-transgenic mouse line (see Materials and Methods), in which tdTomato-labeled pre-BötC glutamatergic neurons were completely distinct from GFP-labeled GABAergic and antibody-labeled glycinergic neurons (*n* = 4 mice; Fig. 4*A*).

*In situ* hybridization for VgluT2 mRNA (Stornetta et al., 2003b; Gray et al., 2010; Tupal et al., 2014) has demonstrated that subpopulations of mouse Dbx1-derived and mouse/rat Sst-expressing pre-BötC neurons are glutamatergic. In our Cre-driven Dbx1-tdTomato line, produced from a different Dbx1-Cre driver line than used previously [i.e., a tamoxifen-inducible Dbx1-Cre line in the studies (Gray et al., 2010; Tupal et al., 2014) examining neurotransmitter phenotype of Dbx1-derived neurons], we confirmed that the majority (98.2%, *n* = 616 of 627 from three mice) of tdTomato-labeled pre-BötC neurons coexpressed glutaminase (Fig. 3*B*). We also verified in our Sst-tdTomato line that the majority of tdTomato-labeled pre-BötC neurons (96.2%, *n* = 552 of 574 from three mice) coexpressed glutaminase (Fig. 3*C*, arrows). We also confirmed, by exploiting our VgluT2-tdTomato-GAD67-GFP mouse line, reacted with glycine and Sst antibodies, that only a small fraction (5.0%, *n* = 11 of 218 from two mice) of Sst antibody-labeled pre-BötC neurons were inhibitory neurons (Fig. 4*B*).

### Validation of Sst-Cre line and Sst antibody labeling of pre-BötC glutamatergic and Dbx1-derived neurons

**Figure 5.**
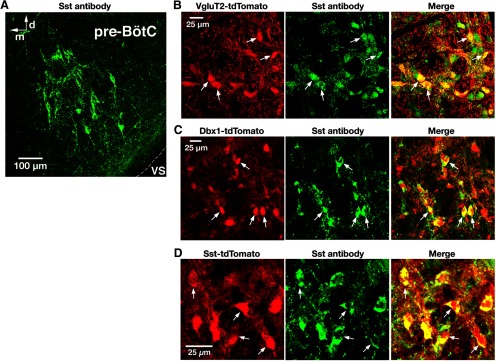
Validation of Sst-Cre line and Sst antibody labeling of pre-BötC glutamatergic and Dbx1-derived neurons. ***A***, Confocal fluorescence microscopy images at low magnification showing Sst antibody-labeled neurons within the pre-BötC in a representative fixed coronal section from adult VgluT2-tdTomato mouse. VS, Ventral surface; d, dorsal; m, medial. ***B***, ***C***, Confocal fluorescence microscopy images of a pre-BötC subregion at higher magnification showing VgluT2 Cre-dependent tdTomato neuronal labeling (red; ***B***), Dbx1 Cre-dependent tdTomato labeling (red; ***C***), and immunolabeling with Sst antibody (green) in pre-BötC neurons. Arrows in the merged image in ***B*** and ***C*** indicate the coexpression of Sst in tdTomato-labeled VgluT2-expressing and Dbx1-derived neurons. ***D***, Confocal images of a pre-BötC subregion in a fixed section from the Sst-tdTomato double-Tg mouse strain illustrating tdTomato neuronal labeling (red) and immunolabeling with Sst antibody (green) colocalized with tdTomato (arrows and merged image). All images have the same dorsomedial anatomical orientation.

We examined Sst antibody labeling in the Sst-tdTomato line and quantitatively verified colabeling with tdTomato in essentially all pre-BötC neurons examined (98.8%, *n* = 1361 of 1378 from four mice; Fig. 5*D*). We also quantified neuronal Sst coexpression in glutamatergic and Dbx1-derived pre-BötC neurons by immunolabeling with Sst antibody in our VgluT2-tdTomato and Dbx1-tdTomato lines. A majority of VgluT2-expressing (78.4%, *n* = 1268 of 1618 from four mice) or Dbx1-derived (72.6%, *n* = 1044 of 1439 from four mice) pre-BötC neurons immunolabeled for Sst (Fig. 5*A–C*).

### Optogenetic inhibition of pre-BötC inspiratory neurons *in vitro*

**Figure 6.**
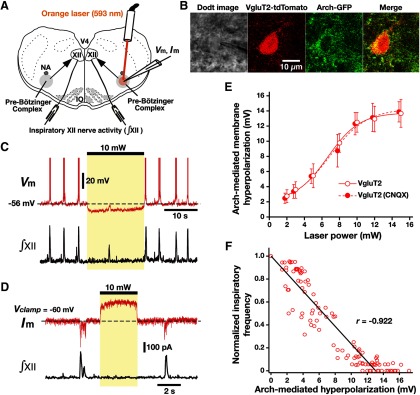
Optogenetic inhibition of rhythmically active Arch-expressing pre-BötC inspiratory glutamatergic neurons *in vitro*. ***A***, Overview of experimental *in vitro* neonatal mouse rhythmic slice preparation with unilateral pre-BötC laser illumination (2-15 mW). NA, Nucleus ambiguus; V4, fourth ventricle. ***B***, Two-photon single-optical plane images of pre-BötC glutamatergic neuron targeted for whole-cell recording, showing a Dodt structural image, VgluT2-Cre-mediated tdTomato labeling, and Arch-GFP expression (coexpression confirmed in merged image). ***C***, Current-clamp recording from pre-BötC inspiratory neuron in ***B*** illustrates inspiratory bursting synchronized with integrated inspiratory hypoglossal activity (∫XII). The membrane potential was hyperpolarized by ∼10 mV at 10 mW laser power. This unilateral illumination was sufficient to reduce the frequency and amplitude of ∫XII. ***D***, Under voltage-clamp, the same neuron as in ***C*** exhibited rhythmic inward synaptic currents synchronized with ∫XII, whereas laser illumination (10 mW) induced outward currents of ∼200 pA. ***E***, Summary data showing Arch-mediated hyperpolarization in VgluT2-expressing pre-BötC inspiratory neurons (*n* = 22) was laser power dependent. After blocking fast glutamatergic synaptic transmission with CNQX, laser-induced hyperpolarization of VgluT2-positive pre-BötC inspiratory neurons (*n* = 6) was also laser power dependent, which was not significantly different from the cases with the synaptically coupled network (unpaired *t* test, *p* = 0.938). ***F***, Linear correlation between laser-induced hyperpolarization of VgluT2-positive pre-BötC inspiratory neurons (*n* = 22) and XII inspiratory frequency, showing the monotonic reduction of frequency as a function of membrane hyperpolarization. Data are represented as the mean ± SD.

**Figure 7.**
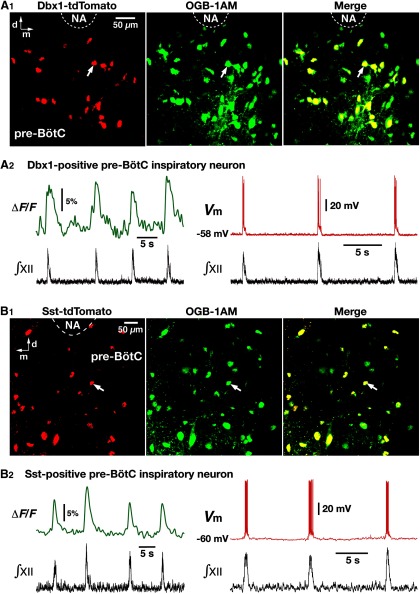
Rhythmically active Dbx1- and Sst-expressing pre-BötC inspiratory neurons identified by Ca^2+^ activity fluorescence imaging and whole-cell patch-clamp recordings. ***A1***, Two-photon single optical plane live images of the pre-BötC region in an *in vitro* neonatal medullary slice from Dbx1-tdTomato Tg mouse line, showing Cre-driven tdTomato reporter protein labeling in Dbx1-derived neurons (red) and Ca^2+^-sensitive fluorescent dye (Oregon Green BAPTA-1 AM, OGB-1AM)-labeled neurons (green). Merged image shows tdTomato-labeled Dbx1-derived neurons colabeled with OGB-1AM by local microinjection. NA, Nucleus ambiguus; d, dorsal; m, medial. ***A2***, Left, Example of rhythmic fluorescence intensity signals from Dbx1-derived pre-BötC inspiratory neuron (***A1***, arrow) illustrating Ca^2+^ transients (top trace) synchronized with electrophysiological signals of inspiratory network activity represented by integrated inspiratory hypoglossal activity (∫XII; bottom trace). Whole-cell current-clamp recording from the same neuron confirms neuronal activity in phase with the ∫XII (right). ***B1***, Two-photon single-optical plane live images of the pre-BötC region in an *in vitro* neonatal medullary slice from Sst-tdTomato Tg mouse line, showing Cre-driven tdTomato reporter protein labeling of Sst neurons (red) and Ca^2+^-sensitive fluorescent dye (OGB-1AM)-labeled neurons (green). Merged image shows tdTomato-labeled Sst-expressing neurons colabeled with OGB-1AM by local microinjection. NA, Nucleus ambiguus; d, dorsal; m, medial. ***B2***, Left, Example of rhythmic fluorescence intensity signals from Sst-expressing pre-BötC inspiratory neuron (***A1***, arrow) with Ca^2+^ transients (top trace) synchronized with electrophysiological signals of inspiratory network activity represented by ∫XII (bottom trace). Right, Inspiratory bursting activity of the same Sst-expressing pre-BötC inspiratory neuron synchronized with ∫XII under whole-cell current-clamp recording.

**Figure 8:**
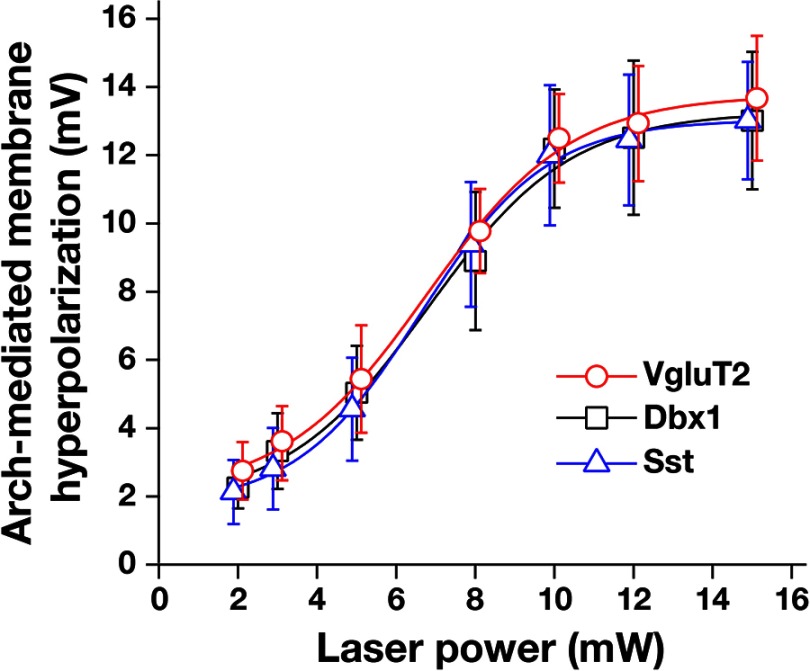
Arch-mediated hyperpolarization in VgluT2-, Dbx1-, and Sst-positive pre-BötC inspiratory neurons. Summary data showing Arch-mediated hyperpolarization in VgluT2-positive (*n* = 22 from Fig. 6), Dbx1-positive (*n* = 28), and Sst-positive (*n* = 19) pre-BötC inspiratory neurons were laser power dependent, and the data fits with a logistic function were not significantly different among these neurons (one-way ANOVA: xhalf: *F*_(2,56)_ = 0.45, *p* = 0.64; slope: *F*_(2,56)_ = 0.74, *p* = 0.48).

We analyzed the efficacy of photoinhibition (593 nm, 2–15 mW) of Arch-expressing, functionally identified pre-BötC inspiratory neurons by whole-cell recordings in rhythmically active *in vitro* neonatal medullary slices from the VgluT2-tdTomato-Arch-GFP, Dbx1-tdTomato-Arch-GFP, and Sst-tdTomato-Arch-GFP Tg mouse lines (Figs. 6–8). These slices effectively isolate the bilateral pre-BötC along with premotor microcircuits for the transmission of the rhythmic inspiratory drive that originates in the pre-BötC and propagates to XII motoneurons (Koizumi et al., 2008), allowing focal perturbations of pre-BötC neuronal activity and simultaneous monitoring of XII inspiratory activity (Fig. 6*A*). By Ca^2+^ activity fluorescence imaging or by randomly patch-clamping tdTomato-labeled neurons imaged with two-photon LSM, we identified subpopulations of rhythmically active pre-BötC inspiratory neurons from whole-cell recording in each triple-Tg line (Figs. 6, 7). The results from random sampling of tdTomato-labeled pre-BötC neurons in each Tg line showed that the ratios of rhythmic inspiratory neurons were 57.1% (*n* = 24 of 42 neurons, *n* = 5 slices) in VgluT2-expressing neurons, 54.3% (*n* = 19 of 35, *n* = 4 slices) in Dbx1-derived neurons, and 35.5% (*n* = 22 of 62, *n* = 6 slices) in Sst-expressing pre-BötC neurons.

In current-clamp recordings (Fig. 6*C*), the membrane potential of VgluT2-expressing pre-BötC inspiratory neurons was maximally hyperpolarized by 12.5 ± 1.3 mV at 10 mW laser power (*n* = 22, unilateral illumination), which was associated with light-induced outward currents of 202 ± 24 pA under voltage-clamp (Fig. 6*D*) with fast kinetics occurring within ∼10 ms, and recovery within ∼20 ms after terminating illumination. Arch-mediated hyperpolarization of VgluT2-, Dbx1-, and Sst-tdTomato positive inspiratory neurons was laser power dependent (Figs. 6*E*, 8). The data fit with a logistic function of laser power versus membrane hyperpolarization in VgluT2-expressing (x value at the logistic curve's midpoint, xhalf = 6.7 ± 0.3, slope = 1.8 ± 0.3, *n* = 22 neurons from four mice), Dbx1-derived (xhalf = 6.8 ± 0.5, slope = 1.8 ± 0.4, *n* = 18 from four mice), and Sst-expressing (xhalf = 6.7 ± 0.2, slope = 1.6 ± 0.2, *n* = 19 from four mice) pre-BötC inspiratory neurons were not significantly different (xhalf: *F*_(2,56)_ = 0.45, *p* = 0.64; slope: *F*_(2,56)_ = 0.74, *p* = 0.48; Fig. 8). Arch-induced outward currents in VgluT2-, Dbx1-, and Sst-positive inspiratory neurons were also confirmed to be laser power dependent (2-15 mW; range for the group data, 24 ± 6.5 to 284 ± 24.3 pA). A component of the membrane hyperpolarization quantified above in neurons within the active respiratory network may result from photoinhibition of Arch-expressing glutamatergic terminals (e.g., from tonically active excitatory neurons outside of the pre-BötC-regulating rhythm generation; Crone et al., 2012). We therefore measured light-induced hyperpolarization after blocking fast glutamatergic synaptic transmission with CNQX (Koshiya and Smith, 1999) in VgluT2-positive pre-BötC inspiratory neurons (logistic function fit: xhalf = 6.9 ± 0.6, slope = 1.9 ± 0.5, *n* = 6; Fig. 6*E*). This logistic function was not significantly different from that obtained with the synaptically coupled active network (VgluT2 neurons without CNQX; unpaired *t* test, *p* = 0.938).

The unilateral pre-BötC illumination was sufficient to reduce the frequency and amplitude of XII inspiratory discharge and also the rhythmic drive potentials of pre-BötC inspiratory neurons (Fig. 6*C*). We therefore analyzed the relationship between Arch-mediated hyperpolarization of VgluT2-expressing pre-BötC inspiratory neurons and XII inspiratory burst frequency (Fig. 6*F*). Inspiratory frequency was reduced monotonically as a function of Arch-mediated membrane hyperpolarization (linear regression fit: slope = −0.077 ± 0.004, *r* = −0.922, *n* = 22 neurons), indicating a voltage-dependent mechanism generating inspiratory rhythm in glutamatergic neurons.

### Perturbations of inspiratory rhythm by bilateral photoinhibition of pre-BötC glutamatergic, Dbx1-derived, and Sst-expressing neurons *in vitro*

**Figure 9.**
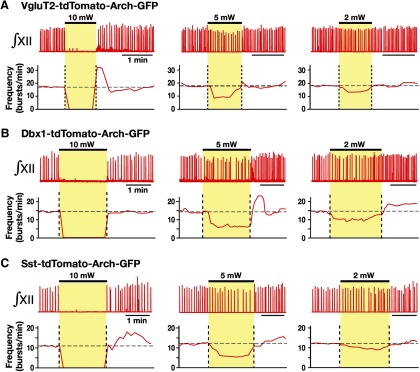
Population-specific Arch-mediated optical perturbations and inhibition of inspiratory rhythm in rhythmically active neonatal mouse *in vitro* medullary slice preparations. ***A–C***, Experiments from VgluT2-tdTomato-Arch-GFP (***A***), Dbx1-tdTomato-Arch-GFP (***B***), and Sst-tdTomato-Arch-GFP (***C***) triple-Tg mouse lines. To systematically analyze the relationship between steady-state frequency and laser power (2-10 mW), we applied single epochs of continuous orange (593 nm) laser illumination (1-2 min). Representative traces of inspiratory network activity monitored by integrated XII nerve (∫XII) recordings, illustrating laser-induced perturbations of inspiratory frequency during bilateral pre-BötC illumination (2, 5, and 10 mW). In all cases, laser application caused rapid and reversible reductions of inspiratory frequency (time-based moving median, solid red lines in panels below the ∫XII traces in ***A***, ***B***, and ***C***) in a laser power-dependent manner with the complete cessation of the inspiratory rhythm at the maximum applied power (10 mW).

**Figure 10.**
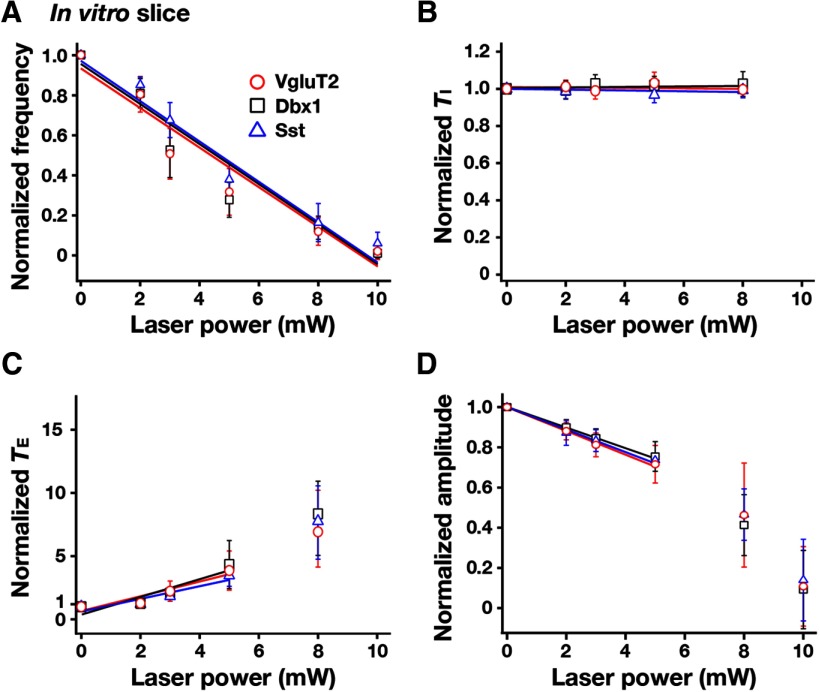
Summary of bilateral pre-BötC optical perturbations of inspiratory frequency, amplitude of inspiratory XII activity, and *T*_I_ and *T*_E_ in rhythmically active *in vitro* slice preparations from VgluT2-Arch-GFP (*n* = 12), Dbx1-Arch-GFP (*n* = 10), and Sst-tdTomato-Arch-GFP (*n* = 10) Tg lines. ***A***, Laser application significantly reduced inspiratory frequency monotonically in a laser power-dependent manner in all cases. These frequency–power relations were not statistically different among VgluT2-, Dbx1-, and Sst-expressing neuronal populations (one-way ANOVA: *F*_(2,29)_ = 0.71, *p* = 0.499). ***B***, *T*_I_ did not significantly change during laser application (*F*_(3,30)_ = 1.72, *p* = 0.182). ***C***, *T*_E_ increased in a laser power-dependent manner, which accounts for the progressive reductions of inspiratory frequency. ***D***, The amplitude of XII inspiratory bursts was reduced monotonically as a function of laser power compared with the control mice (data not shown; *F*_(3,30)_ = 235.77, *p* < 0.0001; Tukey’s test, *p* < 0.01), but these relations (over the power range with nonzero frequency values, see regression lines in ***D***) were not statistically different among the three Arch-expressing lines (Tukey’s test, nonsignificant). Data are represented as the mean ± SD.

We analyzed perturbations of inspiratory frequency, inspiratory/expiratory phase durations, and amplitude of XII inspiratory motor output during continuous laser illumination (593 nm, 2-10 mW, 1-2 min) of the pre-BötC bilaterally in the rhythmically active slices from VgluT2-tdTomato-Arch-GFP (Fig. 9*A*), Dbx1-tdTomato-Arch-GFP (Fig. 9*B*), and Sst-tdTomato-Arch-GFP (Fig. 9*C*) Tg lines. To systematically analyze steady-state frequency versus laser power relations, we applied single epochs of continuous illumination (laser power varied 2-10 mW and allowed for recovery from each epoch before changing power), which demonstrated a rapid and reversible reduction in XII inspiratory frequency as a function of power with termination of XII motor output at 10 mW, which is consistent with the near-maximal hyperpolarization of pre-BötC neurons found at 10 mW power (above). The normalized inspiratory burst frequency versus laser power relations obtained with photoinhibition of pre-BötC VgluT2-expressing (linear regression fit: *r* = −0.925 ± 0.011, slope = −0.098 ± 0.013, *n* = 12 slices), Dbx1-derived (*r* = −0.920 ± 0.010, slope = −0.099 ± 0.014, *n* = 10), or Sst-expressing (*r* = −0.938 ± 0.014, slope = −0.099 ± 0.009, *n* = 10) neuronal populations were not statistically different (*F*_(2,29)_ = 0.71, *p* = 0.499; Fig. 10*A*). In control experiments conducted in slices from the VgluT2-, Dbx1-, or Sst-tdTomato strains (*n* = 2 each), there were no perturbations of frequency, phase durations, or inspiratory burst amplitudes as a function of laser power (2-10 mW; paired *t* test, *p* > 0.05). The reduction in frequency in slices from Arch-expressing strains was due to prolongation of *T*_E_ without significant changes of *T*_I_ (Fig. 10*A–C*). The linear regression fits of *T_I_* versus laser power were not statistically different from the control mice (see above) and also were not significantly different among the three Arch-expressing strains (*F*_(3,34)_ =1.72, *p* = 0.182; Fig. 10*B*). There was a significant reduction in the amplitude of XII inspiratory bursts as a function of laser power compared with the control mice (*F*_(3,34)_ = 235.77; *p* < 0.0001; Tukey’s test, *p* < 0.01), but there was no significant difference among the Arch-expressing strains (Tukey’s test, nonsignificant; Fig. 10*D*). The reduction in the amplitude of XII inspiratory activity is likely due to inhibition of the subpopulation of inspiratory XII premotor neurons known to be located within the pre-BötC (Koizumi et al., 2008), which probably contributes a component of the inspiratory synaptic drive to XII motoneurons. In general, these results suggest that glutamatergic, Dbx1-derived, and Sst-expressing neurons are functionally equivalent in generating the inspiratory rhythm *in vitro*.

**Figure 11.**
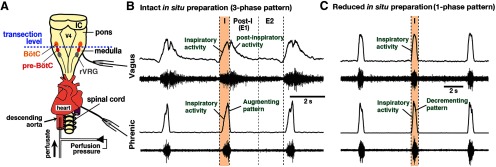
Representative activity patterns of central vagus and phrenic nerves recorded from the intact and the reduced *in situ* arterially perfused brainstem-spinal cord preparations of adult VgluT2-tdTomato-Arch-GFP Tg mice. ***A***, Dorsal view of the intact *in situ* perfused brainstem-spinal cord preparations, also showing the transection level at the rostral boundary of the pre-BötC to obtain the reduced *in situ* preparations. V4, Fourth ventricle; IC, inferior colliculus. ***B***, The intact *in situ* preparations generate a normal three-phase rhythmic pattern with inspiratory (*I*), postinspiratory (E1), and later expiratory (E2) phases. Each panel shows raw (bottom trace) and integrated (top trace) vagus and phrenic motor nerve discharge. The vagus nerves exhibit robust postinspiratory activity as well as inspiratory activity starting before phrenic inspiratory activity. Phrenic inspiratory discharges have an augmenting pattern. ***C***, The physically reduced *in situ* preparations generate a reduced respiratory pattern with one (active)-phase inspiratory rhythmic motor output, generate no postinspiratory activity in the vagus nerve, exhibit the onset of inspiratory activity coincident with the onset of phrenic inspiratory activity, and generate phrenic inspiratory discharges that are converted to a decrementing neonatal slice-like pattern in the adult system.

**Figure 12.**
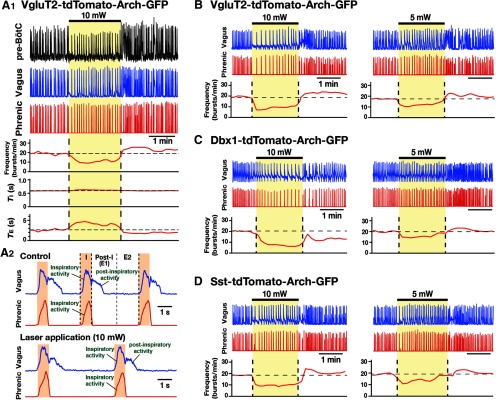
Population-specific optogenetic inhibition of pre-BötC glutamatergic, Dbx1-derived, or Sst-expressing neurons in adult brainstem-spinal cord preparations *in situ*. ***A–D***, Experiments from VgluT2-tdTomato-Arch-GFP (***A***, ***B***), Dbx1-tdTomato-Arch-GFP (***C***), and Sst-tdTomato-Arch-GFP (***D***) mouse lines. ***A1***, Representative traces of simultaneous extracellular recordings of integrated pre-BötC inspiratory neuronal population activity (top trace), as well as integrated inspiratory and postinspiratory activities recorded from vagus nerve (middle trace; represented in ***A2*** with expanded time base) and inspiratory activities from phrenic nerves (bottom trace), with laser-induced effects on inspiratory frequency and *T*_I_ and *T*_E_ (solid red lines; time-based moving median). Bilateral pre-BötC illumination (10 mW, 2 min) caused a rapid and reversible reduction (∼50% in this example) of the frequency of integrated inspiratory activity. The decrease in frequency was due to the prolongation of *T*_E_ without significant changes of *T*_I_. Simultaneous extracellular recordings of pre-BötC inspiratory population activity showed perturbations of inspiratory frequency. ***A2***, The three-phase organization of the respiratory pattern [with inspiratory, postinspiratory (E1), and later expiratory (E2) phases] was not perturbed during bilateral pre-BötC illumination (bottom trace), when compared with the organization before laser application (top trace). ***B–D***, Representative traces of inspiratory and postinspiratory vagus activities, and inspiratory phrenic activity with laser-induced perturbations of inspiratory frequency during bilateral pre-BötC laser illumination (5 and 10 mW). Inspiratory frequency was reduced significantly in a laser power-dependent manner in all cases. The frequency of inspiratory activity was maximally reduced by ∼50% in all cases at 10 mW, but was not eliminated in these *in situ* preparations.

**Figure 13.**
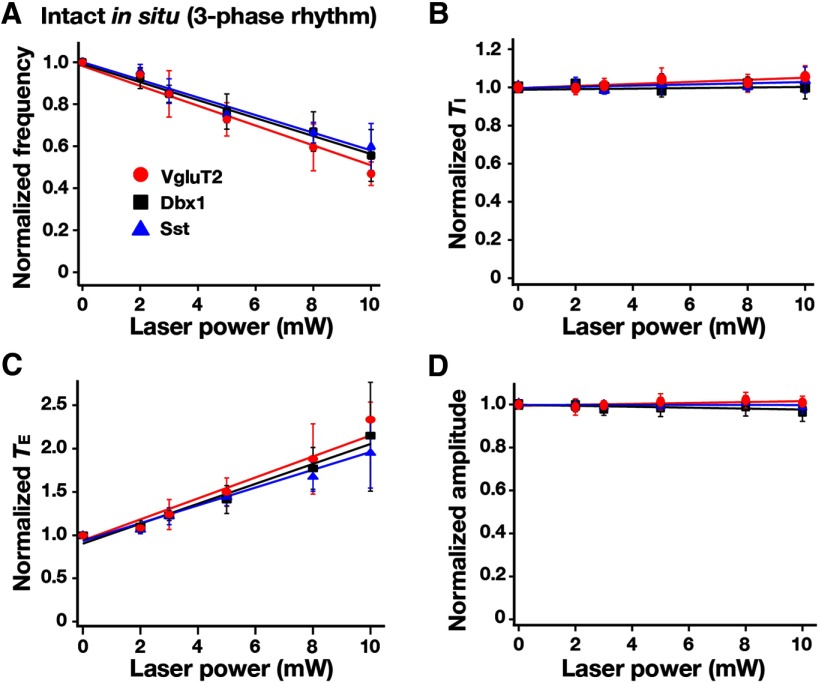
Summary of bilateral pre-BötC optical perturbations of inspiratory frequency, amplitude of inspiratory phrenic activity, and *T*_I_ and *T*_E_ in the intact *in situ* preparations from VgluT2-Arch-GFP, Dbx1-Arch-GFP, and Sst-tdTomato-Arch-GFP (*n* = 8 each) Tg lines. ***A***, Laser application significantly reduced inspiratory frequency monotonically in a laser power-dependent manner in all cases. These frequency–power relations were not statistically different among VgluT2-, Dbx1-, and Sst-expressing neuronal populations (one-way ANOVA: *F*_(2,21)_ = 1.51, *p* = 0.246). ***B***, *T*_I_ did not significantly change (*F*_(3,22)_ = 0.69, *p* = 0.568). ***C***, *T*_E_ increased in a laser power-dependent manner in all cases, which accounts for the progressive reductions in inspiratory frequency. ***D***, The amplitude of phrenic inspiratory bursts was not significantly changed during laser application (*F*_(3,22)_ = 0.89, *p* = 0.462). Data are represented as the mean ± SD.

**Figure 14.**
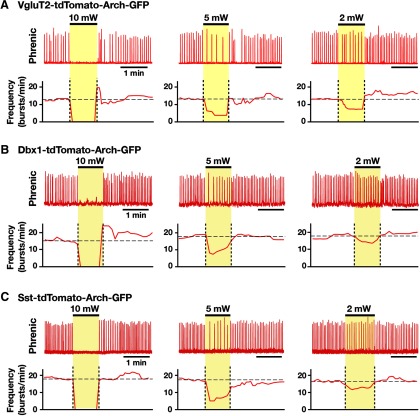
Population-specific Arch-mediated inhibition of pre-BötC glutamatergic, Dbx1-derived, and Sst-expressing neurons in the reduced *in situ* brainstem-spinal cord preparations. ***A–C***, Experiments from adult VgluT2-tdTomato-Arch-GFP (***A***), Dbx1-tdTomato-Arch-GFP (***B***), and Sst-tdTomato-Arch-GFP (***C***) mouse lines. The reduced *in situ* preparation, with transection at the rostral boundary of the pre-BötC, generates a reduced respiratory pattern that is represented by phrenic nerve activity with one (active)-phase (inspiratory but no postinspiratory phase activity; Fig. 11*C*) and is similar to that generated in the *in vitro* neonatal slice preparations. Representative traces of integrated phrenic activities and laser-induced perturbations of inspiratory frequency during bilateral pre-BötC laser illumination (2, 5, and 10 mW), which caused a rapid and reversible laser power-dependent reduction of the inspiratory frequency with complete cessation of the inspiratory rhythm at the maximum applied power (10 mW).

**Figure 15.**
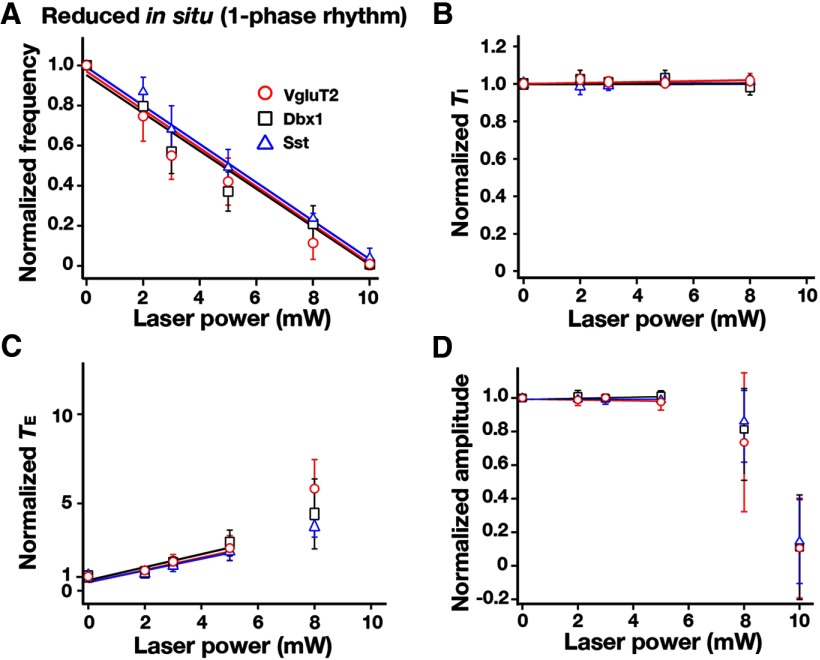
Summary of bilateral pre-BötC optical perturbations of inspiratory frequency, the amplitude of inspiratory phrenic activity, and *T*_I_ and *T*_E_ in the reduced *in situ* preparations from VgluT2-Arch-GFP, Dbx1-Arch-GFP, and Sst-tdTomato-Arch-GFP (*n* = 6 each) Tg lines. ***A***, Laser application significantly reduced inspiratory frequency monotonically in a laser power-dependent manner in all cases. These frequency–power relations were not statistically different among VgluT2-, Dbx1-, and Sst-expressing neuronal populations (one-way ANOVA: *F*_(2,15)_ = 0.60, *p* = 0.564). ***B***, *T*_I_ did not significantly change (*F*_(3,16)_ = 0.56, *p* = 0.649). ***C***, *T*_E_ increased in a laser power-dependent manner, which accounts for the progressive reductions of inspiratory frequency. ***D***, The amplitudes of phrenic inspiratory bursts were not significantly changed (*F*_(3,16)_ = 0.66, *p* = 0.589). Data are represented as the mean ± SD.

**Figure 16.**
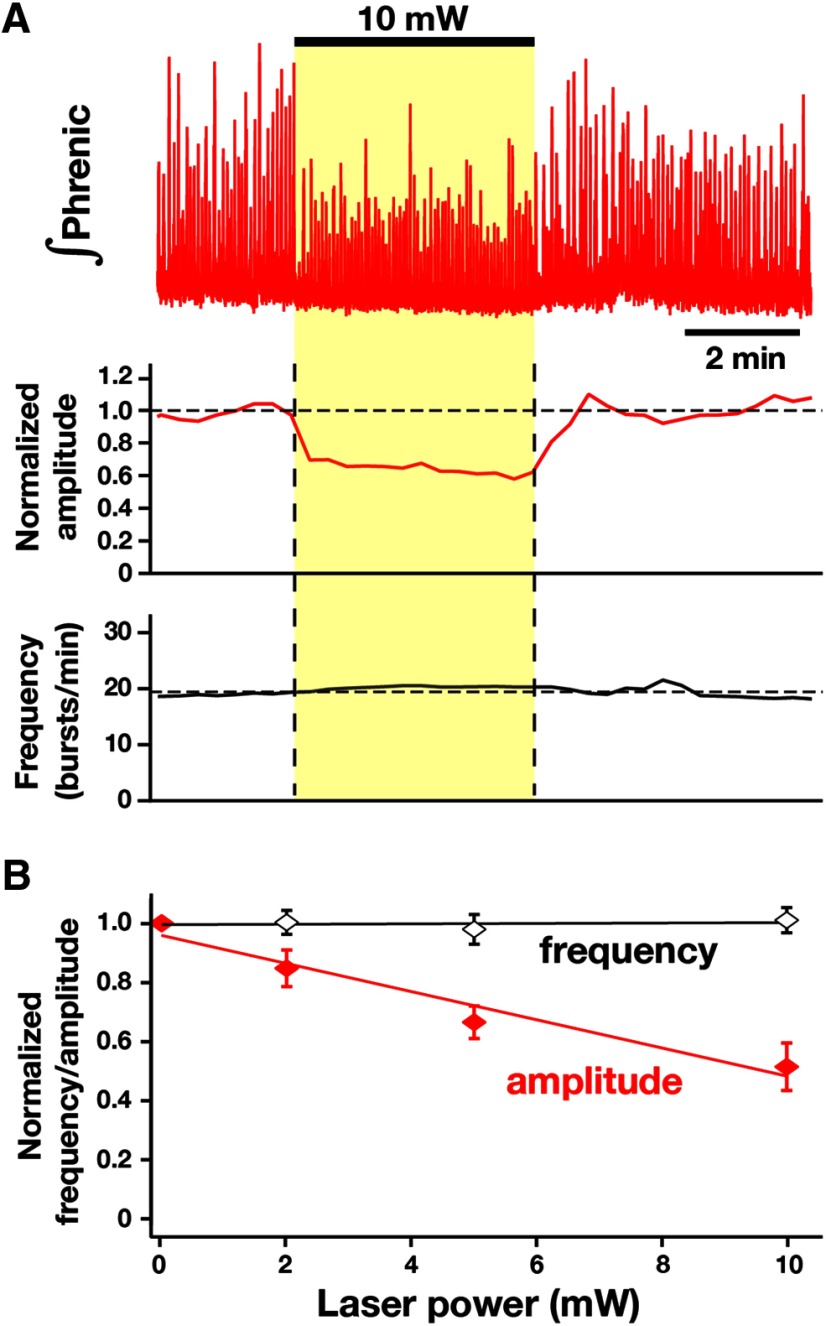
Site specificity in the ventrolateral respiratory column of laser-induced perturbations of the inspiratory rhythm in the intact *in situ* preparations from VgluT2-tdTomato-Arch-GFP mice. ***A***, Representative traces of integrated phrenic inspiratory activity (top trace) illustrating a reduction of inspiratory burst amplitude without perturbations of inspiratory frequency by laser illumination (10 mW) in the rVRG region ∼200 µm caudal to the caudal boundary of the pre-BötC. ***B***, Summary data from five *in situ* preparations showing that, in contrast to when orange light is applied to the pre-BötC region, laser application to the rVRG (2-10 mW) did not perturb inspiratory frequency compared with the frequency before laser application (paired *t* test, *p* = 0.607 at 10 mW), but significantly reduced the amplitude of integrated phrenic inspiratory activity compared with averaged amplitudes before application of the laser (paired *t* test: 2 mW, *p* = 0.016; 5 mW, *p* = 0.0012; 10 mW, *p* = 0.00017), as predicted for the partial inhibition of rVRG bulbospinal excitatory inspiratory neurons. These results indicate that laser illumination specifically in the pre-BötC is required for perturbations of the inspiratory rhythm. Data are represented as the mean ± SD.

### Perturbations of inspiratory rhythm by photoinhibition of pre-BötC glutamatergic, Dbx1-derived, and Sst-expressing neurons *in situ*

We extended the optogenetic experiments to the more intact respiratory network in adult mouse *in situ* brainstem-spinal cord preparations that generate a normal three-phase rhythmic pattern (Fig. 11*B*), produced by more complex circuit interactions, involving the pons, BötC, pre-BötC, and rVRG (Smith et al., 2007), and not operating within the isolated pre-BötC *in vitro*. We analyzed perturbations of the respiratory rhythm and motor output pattern of phrenic and vagus nerve activity (the latter monitoring both inspiratory and postinspiratory activity), and in some experiments with simultaneous recordings of pre-BötC inspiratory neuron population activity (Fig. 12*A*), during continuous bilateral illumination (593 nm, 2–10 mW, 1–2 min) of the pre-BötC in VgluT2-tdTomato-Arch-GFP (Fig. 12*A*,*B*), Dbx1-tdTomato-Arch-GFP (Fig. 12*C*), and Sst-tdTomato-Arch-GFP (Fig. 12*D*) Tg mouse preparations. In all cases, bilateral pre-BötC photoinhibition significantly reduced the steady-state inspiratory frequency in a laser power-dependent manner with a maximal reduction of ∼50% at 10 mW (compared with the frequency before laser application; paired *t* test: VgluT2, *p* = 0.0002; Dbx1, *p* = 0.0003; Sst, *p* = 0.0003; Fig. 13*A*) with no further reductions of inspiratory frequency at higher power up to 15 mW. The monotonic relations between normalized frequency and laser power obtained by linear regression in VgluT2-tdTomato-Arch-GFP (*r* = −0.969 ± 0.011, slope = −0.048 ± 0.012, *n* = 8), Dbx1-tdTomato-Arch-GFP (*r* = −0.964 ± 0.013, slope = −0.046 ± 0.009, *n* = 8), and Sst-tdTomato-Arch-GFP (*r* = −0.964 ± 0.015, slope = −0.045 ± 0.012, *n* = 8) preparations were not significantly different for the three strains (*F*_(2,21)_ = 1.51, *p* = 0.246; Fig. 13*A*). The simultaneous extracellular recordings of pre-BötC inspiratory population activity confirmed laser power-dependent perturbations of the frequency of pre-BötC inspiratory activity in all three strains (VgluT2, *n* = 4; Dbx1, *n* = 3; Sst, *n* = 3). Similar to *in vitro* experiments, the observed decrease of frequency was due to the prolongation of *T*_E_ without significant changes in *T*_I_ (Figs. 12*A*, 13*A–C*). The linear regression fits of *T*_I_ versus laser power were not statistically different from those of the control mice (see experimental procedures), and also were not significantly different among three Arch-expressing strains (*F*_(3,23)_ = 0.69, *p* = 0.568; Fig. 13*B*). The amplitudes of inspiratory phrenic and vagus nerve activities were not perturbed during bilateral pre-BötC illumination, with no significant differences among the three strains (phrenic: *F*_(3,23)_ = 0.89, *p* = 0.462; vagus: *F*_(3,23)_ = 0.72, *p* = 0.551; Fig. 13*D*). The basic three-phase organization of the respiratory pattern was also not disturbed (Fig. 12*A2*).

In these *in situ* experiments, the maximum applied laser power (≥10 mW) was unable to terminate the inspiratory rhythm, in contrast with the results obtained with *in vitro* preparations, which is likely due to insufficient Arch-mediated hyperpolarization of pre-BötC neurons (see Discussion). We, therefore, reduced the excitation state of pre-BötC circuits by removing pontine and RTN excitatory inputs via transection of the perfused preparations at the rostral boundary of the pre-BötC, which converted the three-phase operation of the network to a reduced pattern with a one-phase (active phase) inspiratory pattern (Fig. 11*C*) similar to that generated *in vitro*, as previously described (Smith et al., 2007). This preparation allowed rigorous comparison of glutamatergic, Dbx1-derived, and Sst-expressing (*n* = 6 each) neuronal population roles in rhythm generation intrinsic to pre-BötC circuits in the reduced adult versus neonatal systems. Epochs (1-2 min for steady-state perturbation) of bilateral pre-BötC photoinhibition reduced inspiratory frequency as a function of laser power and eliminated respiratory rhythm at 10 mW in all cases (Fig. 14*A–C*). The monotonic relations between the normalized frequency and laser power obtained by linear regression analysis for VgluT2-expressing (*r* = −0.934 ± 0.017, slope = −0.098 ± 0.008), Dbx1-derived (*r* = −0.929 ± 0.013, slope = −0.097 ± 0.009), and Sst-expressing (*r* = −0.940 ± 0.016, slope = −0.098 ± 0.008) populations were not significantly different (*F*_(2,15)_ = 0.60, *p* = 0.564; Fig. 15*A*), and were essentially identical to those obtained *in vitro* (reduced *in situ* vs. *in vitro*, unpaired *t* test: VgluT2, *p* = 0.958; Dbx1, *p* = 0.886; Sst, *p* = 0.889). As *in vitro*, the observed decrease in frequency was due to the prolongation of *T*_E_ without significant changes in *T*_I_ (Fig. 15*A–C*). However, the amplitudes of both vagus and phrenic inspiratory activity were not significantly perturbed by photoinhibition *in situ* (Fig. 15*D*). The effects of laser application on *T*_I_ and the amplitude of phrenic inspiratory activity were not significantly different from those of the control mice, and also were not different for the three Arch-expressing strains (*T*_I_: *F*_(3,17)_ = 0.56, *p* = 0.649; amplitude: *F*_(3,17)_ = 0.66, *p* = 0.589).

### Site specificity in the ventrolateral respiratory column of laser-induced perturbations of the inspiratory rhythm in the intact *in situ* preparations

We verified site-specificity of light-induced perturbations of the rhythm in the ventrolateral respiratory column (VRC) by systematically repositioning the optical cannula bilaterally along the VRC from the caudal end of the pre-BötC into the rVRG (*n* = 5) or from the rostral end of the pre-BötC into the BötC (*n* = 3) in the intact *in situ* preparations from VgluT2-tdTomato-Arch-GFP mice. Illumination (2–10 mW) in the rVRG at sites only 200 µm caudal to the boundary of the pre-BötC did not significantly perturb inspiratory frequency in comparison to the frequency before laser application (paired *t* test: 2 mW, *p* = 0.891; 5 mW, *p* = 0.482; 10 mW, *p* = 0.607), but significantly reduced the amplitude of phrenic inspiratory activity (paired *t* test: 2 mW, *p* = 0.016; 5 mW, *p* = 0.0012; 10 mW, *p* = 0.00017; Fig. 16) as predicted for the partial inhibition of rVRG bulbospinal inspiratory premotor neurons known to be located in this region (Stornetta et al., 2003a). Laser illumination in the BötC region 200 µm away from the pre-BötC rostral boundary also did not significantly perturb inspiratory frequency (paired *t* test, *p* = 0.7389 at 10 mW).

## Discussion

Defining the functional roles of specific neuron populations in brainstem respiratory circuits, particularly in the pre-BötC, is required to unravel the origin and mechanisms of breathing in mammals. A central problem has been to demonstrate that the pre-BötC glutamatergic neuron population is rhythmogenic, which has not been possible without applying techniques to specifically manipulate the activity of these neurons. By using optogenetic approaches combined with electrophysiological analysis, we demonstrate such a rhythmogenic role of pre-BötC glutamatergic neurons at the population level, and provide evidence that a neuronal voltage-dependent mechanism operating in the pre-BötC excitatory circuits underlies rhythmogenesis. We have established this mechanism in different functional and developmental states of respiratory circuits, including structurally reduced conditions in the neonate and adult, as well as the behaviorally normal activity state that generates the three-phase rhythmic pattern in the more intact system. Our results therefore suggest in general that glutamatergic neurons, and neurons derived from Dbx1-expressing progenitors or neurons expressing Sst in the pre-BötC have a functionally and mechanistically similar rhythmogenic property.

### Experimental validation of Cre-driver and Cre-driven optogenetic mouse lines

We established three different Tg lines with Cre-driven Arch expression selectively in glutamatergic, Dbx1-derived, or Sst-expressing neurons to allow cell type-specific manipulation of population activity (Deisseroth et al., 2006; Yizhar et al., 2011; Madisen et al., 2012). To test the causal roles of these populations optogenetically, it was crucial to validate these Tg lines for Cre expression and Cre-driven Arch expression patterns, to establish the efficacy of Arch-mediated photoinhibition at the pre-BötC cellular and circuit levels, and to determine the site specificity of the optical perturbations.

We initially validated the Cre-driver mouse lines by a combination of tdTomato reporter expression and immunohistochemical analyses, particularly in the pre-BötC region, given the possibility of regional variation in Cre expression for each transgenic line. In the VgluT2-Cre-tdTomato line, we confirmed tdTomato expression extensively in pre-BötC neurons and that essentially all the tdTomato-labeled neurons (98.5%) expressed glutaminase antibody, which is a necessary (although not exclusive) condition to be satisfied for a glutamatergic phenotype. Additionally, we demonstrated no overlap of tdTomato neuronal expression with GABAergic and glycinergic neurons in the VgluT2-tdTomato-GAD67-GFP line. In the Sst-Cre-tdTomato line, we confirmed that pre-BötC Sst neurons were colabeled by glutaminase (96.2%) or Sst antibodies (98.8%). Since Dbx1 is only transiently expressed in progenitor cells, we could not directly analyze Dbx1 expression in our Dbx1-Cre-tdTomato line. However, evidence consistent with their glutamatergic neuronal fate was obtained by glutaminase antibody colabeling with tdTomato in a majority (98.2%) of Dbx1-derived pre-BötC neurons.

In the VgluT2-tdTomato-Arch-GFP, Dbx1-tdTomato-Arch-GFP, and Sst-tdTomato-Arch-GFP lines, we verified broad and uniform Arch expression in pre-BötC neurons. We directly validated activity silencing of functionally identified pre-BötC inspiratory neurons as well as laser power-dependent Arch-mediated outward currents/membrane hyperpolarization during whole-cell recordings in the *in vitro* preparations from individual strains. There were no significant quantitative differences in the efficacy of Arch-mediated hyperpolarization among VgluT2-, Dbx1-, and Sst-expressing pre-BötC inspiratory neurons. Additionally, optogenetic inhibition observed in extracellular recordings of pre-BötC population activity in the adult *in situ* preparations confirmed the cell population-level perturbations within the pre-BötC in all three Tg lines. Satisfying these conditions enabled us to comparatively analyze the roles of each VgluT2-, Dbx1-, and Sst-expressing neuronal population in respiratory rhythm and motor pattern generation. Furthermore, the perturbations of inspiratory rhythm were demonstrated to be site specific. Laser illumination in adjacent reticular formation respiratory regions, even 200 µm away from the pre-BötC boundaries, did not perturb the rhythm. Remarkably, directing the light at the pre-BötC in the adult *in situ* brainstem-spinal cord preparations reduced inspiratory frequency without changing the amplitude of phrenic nerve inspiratory activity, whereas the converse occurred when the light was directed only slightly more caudally in the rVRG, where bulbospinal inspiratory premotoneurons are concentrated. These results provide important additional evidence that the inspiratory rhythm and amplitude are controlled by largely different subsets of glutamatergic neurons, in addition to the evidence that the rhythm is mediated by the activity of glutamatergic neurons located within the pre-BötC.

In the Dbx1-tdTomato-Arch-GFP Tg line, Arch is expressed not only in Dbx1-derived neurons, but also in Dbx1-derived glia (Gray et al., 2010). Recently, astrocytes in the pre-BötC region have been shown to be potentially involved in respiratory modulation (Okada et al., 2012). The pre-BötC glial population could therefore contribute to the Arch-mediated perturbations of respiratory rhythm observed in the Dbx1-Cre-Arch Tg line. This possibility is not excluded and requires further investigation of Arch-mediated effects on glial function and any associated perturbation of pre-BötC neural circuit activity.

### Rhythmogenic properties of pre-BötC glutamatergic neurons revealed by optical control

The pre-BötC contains heterogeneous rhythmic interneuron populations of glutamatergic (Gray et al., 2010; Koizumi et al., 2013; Picardo et al., 2013), glycinergic (Winter et al., 2009; Morgado-Valle et al., 2010), and GABAergic (Kuwana et al., 2006) phenotypes. Results from a variety of experimental approaches, including electrophysiological/imaging studies (Koshiya and Smith, 1999; St-John et al., 2009; Koizumi et al., 2013), single-cell RT-PCR/*in situ* hybridization analyses (Stornetta et al., 2003b; Koizumi et al., 2010), and genetic ablation of VgluT2-expressing neurons (Wallen-Mackenzie et al., 2006), suggest that the bilaterally distributed, interconnected (Stornetta et al., 2003b; Bouvier et al., 2010; Koizumi et al., 2013) pre-BötC glutamatergic neurons are the rhythmogenic kernel population, although previously it has not been possible to test this hypothesis directly. Several models (Butera et al., 1999a,b; Smith et al., 2000; Rubin et al., 2009) of the dynamic operation of pre-BötC circuits postulate that a subpopulation of glutamatergic neurons have voltage-dependent cellular properties that provide intrinsic mechanisms for dynamic frequency control under conditions where pre-BötC circuits are isolated, as in neonatal slices *in vitro* or structurally reduced *in situ* preparations. Frequency regulation is primarily via *T*_E_, reflecting the kinetics of postinspiratory biophysical processes operating during the expiratory interval in these models. Our results with graded photoinhibition directly demonstrate this frequency control that is highly correlated with the progressive hyperpolarization of glutamatergic neurons, and, importantly, show that selective silencing of these neurons eliminates rhythmogenesis *in vitro*, establishing a causal role. A similar control of frequency and termination of rhythmogenesis occurs when the pre-BötC is operating in the reduced adult *in situ* preparations. Thus, these properties are intrinsic to pre-BötC excitatory circuits in both neonates and adults. In the intact adult *in situ* preparations, we also demonstrated optical control of frequency, also primarily via *T*_E_, during the normal three-phase respiratory pattern, as predicted by theoretical models (Rubin et al., 2009), although the maximum applied laser power was unable to terminate the inspiratory rhythm. In these intact preparations, the pre-BötC respiratory network receives excitatory synaptic inputs from multiple sources, including from the pons and RTN (Bochorishvili et al., 2012), which are required for three-phase rhythmic pattern generation (Smith et al., 2007). These excitatory synaptic inputs in the more intact preparations contribute to larger-amplitude rhythmic drive potentials (∼20 mV) in pre-BötC circuits (Richter and Smith, 2014) and likely necessitate larger hyperpolarization levels to completely disrupt rhythm generation. However, we do not exclude the possible recruitment and participation in rhythm generation of other pre-BötC glutamatergic neurons that receive excitatory inputs in the intact *in situ* preparations, which remains to be clarified.

We note that photoinhibition of Arch-expressing glutamatergic presynaptic terminals on pre-BötC neurons might contribute to the correlation between frequency and light-induced neuronal hyperpolarization. The similar relation between laser power and hyperpolarization obtained when fast glutamatergic synaptic interactions were blocked, at least *in vitro*, largely rules out such a contribution.

### Common rhythmogenic property of pre-BötC glutamatergic, Dbx1-derived, and Sst-expressing neuron populations

We established from our comparative optogenetic analysis that pre-BötC glutamatergic, Dbx1-derived, and Sst-expressing neurons in each network state examined are equally effective in attenuating/disrupting rhythm generation in a quantitatively identical voltage-dependent manner, indicating that these neurons have a common rhythmogenic property. We interpret this result to indicate that the critical rhythmogenic kernel of glutamatergic neurons functionally active *in vitro* and *in situ* also have, to a sufficient extent, the Dbx1 descendance and Sst expression phenotype, providing a considerable functional overlap of these phenotypically defined neuron populations. A significant anatomical overlap of these phenotypic populations is implied from our current and previous structural analyses (Stornetta et al., 2003b; Bouvier et al., 2010; Gray et al., 2010). However, we emphasize that these anatomical analyses do not identify rhythmically active neurons and thus, per se, do not predict functional properties. We found that 78% of VgluT2-expressing and 73% of Dbx1-derived pre-BötC neurons coexpress Sst by antibody labeling, and a previous study (Bouvier et al., 2010) showed that 91% of pre-BötC Sst-positive neurons were derived from Dbx1 progenitors. *In situ* hybridization showed that 82% of pre-BötC Dbx1-derived neurons are glutamatergic (Bouvier et al., 2010), consistent with our results (98.2%). Immunohistochemical studies (Stornetta et al., 2003b) show that Sst-immunoreactive neurons in the pre-BötC are essentially glutamatergic (∼98%), which is also consistent with our results (96.2%). We also emphasize that we do not know the exact fraction of the rhythmogenic glutamatergic population that also has the Dbx1 and Sst expression phenotypes. The functional overlap is apparently sufficient to confer the same voltage-dependent rhythmogenic property.

Our results demonstrate that like glutamatergic neurons, optogenetic inhibition of pre-BötC Dbx1-derived or Sst-expressing neuronal populations significantly disrupts inspiratory rhythm generation in both neonatal and adult systems, supporting our general conclusion that these phenotypes are functionally/mechanistically equivalent in rhythm generation. These results are consistent with previous results that genetic ablation or photoablation of pre-BötC Dbx1-derived neurons disrupts inspiratory rhythm in newborn mice (Bouvier et al., 2010; Wang et al., 2014), and we now show that these neurons have a site-specific causal role in rhythm generation in the adult system. Furthermore, our results provide new additional evidence that pre-BötC Sst-expressing neurons are critically involved in respiratory rhythm generation (Tan et al., 2008). Although rhythmically active Sst-expressing neurons were not previously identified, we now establish that a subpopulation of these neurons have inspiratory activity in the neonatal system *in vitro* and in the adult system *in situ*, and we show that Sst-expressing neurons have a voltage-dependent mechanism for frequency control, which has also not been demonstrated previously. The essential role of Sst-expressing neurons in breathing has recently been questioned from experiments showing that the elimination of glutamate release from Sst-expressing pre-BötC mouse neurons by genetic ablation of VgluT2, which induces a more general disruption of the connectivity of the respiratory network, had no effect on viability *in vivo* or on respiratory period *in vitro* (Tupal et al., 2014). However, different from short-term silencing of pre-BötC Sst-expressing neurons, the loss of VgluT2 in Sst-expressing neurons during development may have produced a compensatory functional upregulation of other neuron populations or may have been compensated for by the upregulation of another VGluT (e.g., VGluT1 or VGluT3) in Sst-expressing neurons. These interesting developmental plasticity issues of the respiratory neural network remain to be clarified. Our results, also based on Cre-dependent recombination under control of the Sst-Cre transgene, are incongruent with the suggestion that pre-BötC Sst-expressing neurons are not a genetically defined essential population (Tupal et al., 2014).

Previous studies using molecular, cellular electrophysiological, and structural analyses have shown that identified rhythmically active pre-BötC glutamatergic neurons have the appropriate bilateral circuit connectivity and neuronal intrinsic voltage-dependent properties to constitute the rhythmogenic kernel (Koizumi et al. 2013). Our current results support and extend this work by establishing the rhythmogenic role of active glutamatergic neurons at the population level. The optogenetic and electrophysiological approaches used here allowed this role to be verified in all network states examined, both in the neonatal *in vitro* and adult *in situ* systems. Furthermore, we have demonstrated that the pre-BötC glutamatergic, Dbx1-derived, and Sst-expressing neurons have in common a voltage-dependent rhythmogenic property, although the underlying biophysical mechanisms at the cellular and synaptic levels remain to be established.

## Synthesis

The decision was a result of the Reviewing Editor Douglas Bayliss and the peer reviewers coming together and discussing their recommendations until a consensus was reached. A fact-based synthesis statement explaining their decision and outlining what is needed to prepare a revision is listed below. The following reviewer(s) agreed to reveal their identity: Jan-Marino Ramirez

Two reviewers have provided comments on this manuscript and both were very positive about the work. One reviewer raised again the question about the methods for cell counting, suggesting use of a nuclear marker to avoid double-counting and ensure that all staining is associated with a cell body.

My view is that the data are convincing, even if not completely precise, especially since you counted a lot of cells in multiple sections and are using percentages, rather than absolute numbers of cells. It is advised that you make this point for the reader explicitly in the final manuscript, and perhaps reference an editorial on this topic from Saper for JCN (J Comp Neurol. 1996 Jan 1;364(1):5; PMID:8789271).

BTW, below is the relevant section of the Saper JCN policy ststement.

"Another example of a situation in which stereological methods may not be necessary is in determining a rough percentage of a population of cells that are double-labeled because they project to two targets, or stain with two antisera for different neurotransmitters. We agree with Coggeshall and Lekan that estimates of the true size of the populations involved require stereological methods, because profile counting methods contain implicit assumptions about cell size and shape being identical among the different cell types, and this information generally is not available. On the other hand, if the goal is to say that virtually all the cells are double-labeled (or none of them are) or to provide a rough estimate (about one-third of the cells are double-labeled), the biological and technical variability is likely to be much larger than the errors introduced by using potentially biased counting methods. We would accept such semi-quantitative estimates from biased counting methods, but would require that more precise estimates be made by using more precise methods."
